# Investigation on possible resistance of *Candida auris* to irradiation with UVC (254 nm)

**DOI:** 10.17912/micropub.biology.001421

**Published:** 2025-03-20

**Authors:** Anna-Maria Gierke, Blende Demelezi, Martin Hessling

**Affiliations:** 1 Institute of Medical Engineering and Mechatronics, Technische Hochschule Ulm, Ulm, Baden-Wurttemberg, Germany

## Abstract

*Candida auris* is a yeast that is increasingly pathogenic due to increased multiresistance to antimycotics. UV (ultraviolet) radiation is still capable of reducing *C. auris* and offers possible applications in radiation disinfection of surfaces and air. However, it is unknown whether *C. auris* could also develop a resistance against UV radiation. For this reason, the extent to which *C. auris* can develop resistance to UVC radiation (254 nm) over several irradiation cycles is being examined. The yeast is irradiated with a UVC dose of 18.2 mJ/cm ^2^ per cycle, followed by a recovery phase under dark conditions. This irradiation-recovery process was repeated for 16 consecutive cycles, ensuring that the UVC dose of 18.2 mJ/cm ^2^ remained constant throughout all cycles. It was observed that the log reduction (a log 1 reduction corresponds to a 90% reduction) decreased with increasing number of cycles. Resistance development against UVC could be presented.

**Figure 1. 254 nm Inactivation curves of C. auris for 16 irradiation cycles:**
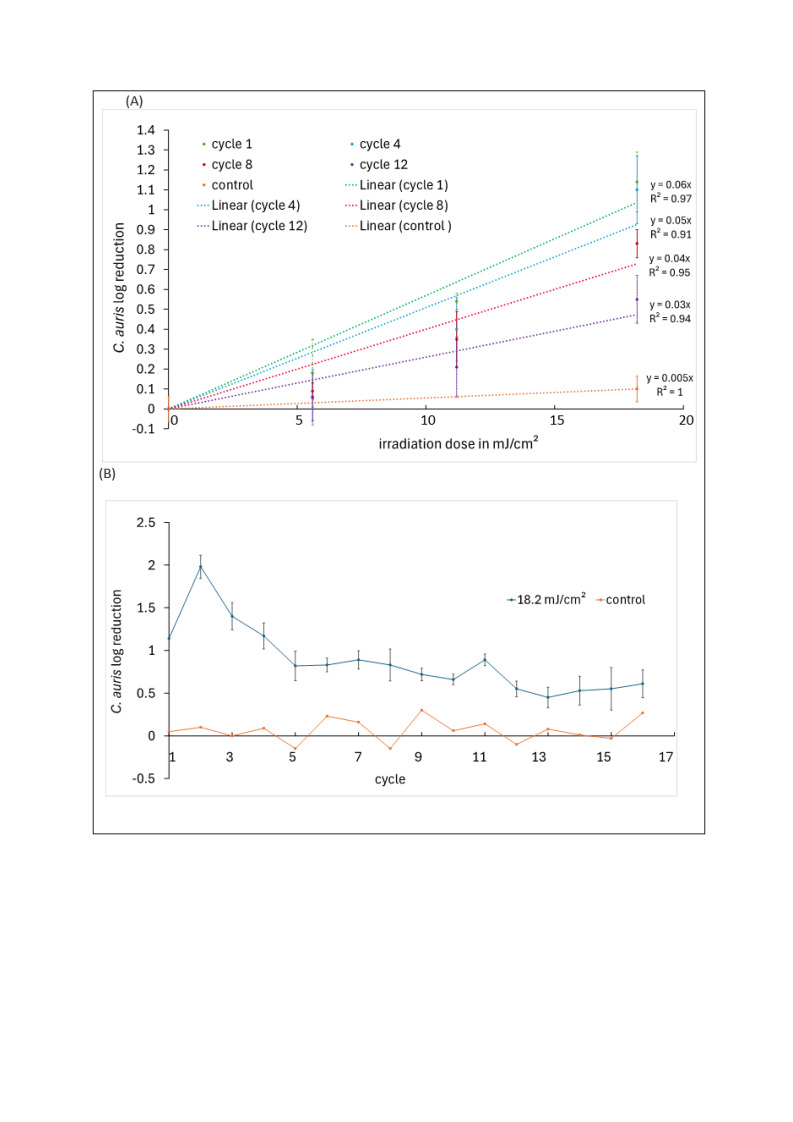
The log reduction represents inactivated fungi in relation to the unirradiated fungi sample, whereby a log 1 reduction corresponds to a 90% reduction of the fungi sample. After each cycle, the yeast could recover under dark conditions. (A) log reduction for different cycles and applied linear fit curves. (B) log reduction over cycles with same irradiation dose (18.2 mJ/cm ^2^ ) are presented for irradiated and unirradiated samples.

## Description

*Candida auris* is an emerging yeast that is particularly lethal for immunocompromised patients [1, 2]. It is increasingly being detected in healthcare facilities and hospitals, with cases of antifungal drug-resistant *Candida auris* on the rise [3–5]. Furthermore, this yeast was listed in the “critical priority group” of the “WHO fungal priority pathogen list” in 2022.

One of the techniques to control *C. auris* is the application of UVC radiation [6, 7]. Irradiation with UVC (200-280 nm) causes DNA (deoxyribonucleic acid) damage [8]. This results in cyclobutane pyrimidine dimers (CPD) and 6-4 photoproducts in DNA, which can lead to mutagenic effects and even cell death [9, 10]. However, there are various repair mechanisms within the microorganisms that can repair these DNA damages [11, 12].

*Escherichia coli* has already been shown to develop a certain resistance to UV radiation over several irradiation cycles [13–16]. Further experiments have been carried out on a small number of bacteria, such as *Staphylococcus aureus* [17–19]. So far, there are no comparable studies on fungi.

Therefore, it is also important to investigate whether a resistance development is possible over time for this yeast. For this reason, this study investigates whether there is any kind of adaptation to an unchanged irradiation dose over 16 cycles of UVC irradiation and recovery.

The suspensions with *C. auris* were irradiated with a dose of 18.2 mJ/cm ^2^ at an UVC wavelength of 254 nm. Figure 1 demonstrates the results of 16 irradiation cycles. Figure 1A presents semi-logarithmically the *C. auris* reduction for different irradiation doses after several cycles of irradiation and recovery. A log 1 reduction dose of 16.7 mJ/cm ^2^ was determined after the 1st cycle, 17.9 mJ/cm ^2^ after the 4th cycle, 25 mJ/cm ^2^ after the 8th cycle and 50 mJ/cm ^2^ following the 12th cycle.

In Figure 1B, the observed log reduction or a fixed 254 nm irradiation dose of 18.2 mJ/cm ^2^ after the different cycles is given. After the first irradiation, a log reduction of 1.23 is observed, which increases further in the 2nd cycle to a log reduction of 1.98. In the subsequent cycles, the log reduction decreased to a log reduction of 0.45 following cycle 13. The results describe the sensitivity of *C. auris* to cyclic irradiation with UVC. It was observed that the log reduction increased in the first irradiation cycles and decreased thereafter. Thus, more fungi survived at the same irradiation dose as the number of cycles increased.

This decrease in log reduction may be attributed to the development of irradiation resistance. As already described by Alcánatara-Díaz et al. and Shibai et al., UV irradiation is a selection pressure exerted on microorganisms [14, 15]. The damage to DNA results in various mutations. UV radiation increases the rate of possible mutations. As a result of selection, only those that are adapted to the new environment can survive and reproduce [15, 12]. Various ideas are compiled as how possible resistance to UV radiation can arise. The concepts describe the activation of individual genes or combined with different genes over several generations, which should result in faster (DNA) repair. Fungi with the best repair mechanisms survive and can multiply [20, 21].

Resistance formation could presumably be observed for *C. auris* . Over the cycles, a lower sensitivity to UV radiation was observed for the yeast. This was also described in the irradiation curves. The slope of the fit curves decreased with increasing number of cycles.

## Methods

*C. auris* (DSM 21092) was cultivated for liquid cultures in modified YEPG (Yeast Extract Peptone Glucose medium - 200 ml glucose (250 g/l), 20 g peptone from casein, 10 g yeast extract per 1,000 ml (pH 6.5) and for agar plates on potato dextrose agar (M129 - 20 g dextrose, 4 g potato extract, (15 g agar) per 1,000 ml; pH 5.6). Cultivation was carried out at 30 °C. For pre-culture, 5 ml liquid medium was cultivated for 24 hours. To carry out the experimental growth and irradiation cycles, 6 subcultures (5 irradiated cultures and 1 control culture) were prepared with a sample of the preculture (1:150). Each main culture consisted of 4 ml total volume. The cultures were placed in 6-well plates and were shaken with a frequency of 170 Hz. The OD _600nm_ (optical density at 600 nm) was controlled regularly until it reached a median OD _600nm_ value of 1.24, just before the stationary phase and the cultures could be removed for the subsequent irradiation.

For irradiation experiments, 2 ml of the cultures were removed and centrifuged at 7,000 rpm for 5 min. After discarding the supernatant, the left pellet was resuspended with phosphate buffered saline (PBS). The washing procedure was repeated. This was followed by a transmission measurement at 254 nm to dilute the washed culture to a transmission of 70-80 % for the 3 mm sample thickness, resulting in a concentration of 1x10 ^7^ colony forming units (CFU)/ml, ensuring uniform irradiation across all layers of the sample. This transmission was measured using a spectrophotometer (SPECORD 250 PLUS double beam spectrophotometer, Analytik Jena GmbH+Co. KG, Jena, Germany) with a 10 mm quartz cuvette.

For irradiation at 254 nm, a low-pressure mercury vapour lamp (TUV 87 15W/G15T8, Philips, Netherlands) was applied as radiation source. An irradiation dose of 18.2 mJ/cm ^2^ (0.7 mW/cm ^2^ ) was chosen after preliminary experiments. After each irradiation, 100 µl samples were taken and, after dilution, plated for subsequent determination of yeast concentration. The irradiation curves were presented semi-logarithmically for different cycles and linear fit curves were added. Using the resulting equation, the log 1 reduction dose for the respective cycles were calculated. In addition, 300 µl from the irradiated sample were taken and mixed with 2,700 µl fresh YEPG medium for the “recovery culturing” of *C. auris* at 30 °C. The ambient room temperature was maintained at 25 °C. The growth period after the initial four cycles ranged between 29 and 36 hours, after which an average recovery cycle duration of 25.5 hours was observed. This corresponds to the starting point of a new cycle. Both, agar plates and cultures of the new cycle remained covered with aluminum foil to avoid possible photoreactivation.
